# Effect of addition of wheat bran hydrolysate on bread properties

**DOI:** 10.1111/1750-3841.17015

**Published:** 2024-03-27

**Authors:** Ali Cingöz, Özlem Akpinar, Abdulvahit Sayaslan

**Affiliations:** ^1^ Department of Food Engineering Tokat Gaziosmanpasa University Tokat Turkey; ^2^ Department of Food Engineering Karamanoğlu Mehmetbey University Karaman Turkey

**Keywords:** bran, bread, hydrothermal pretreatment, starch fractions

## Abstract

Although the addition of bran to bread makes it healthier and more functional, it brings with it some technological problems. One way to eliminate these problems is hydrothermal pretreatment of wheat bran. In this study, five different ratios (10%, 20%, 30%, 50%, and 100%) of hydrolysates from hydrothermal pretreatment of wheat bran (150°C, 30 min) were substituted with dough‐kneading water during dough kneading for bread making. The physical, chemical, functional, textural and important starch fractions of the bread produced were determined. The addition of hydrolysate in different amounts to the dough‐kneading water resulted in similar physical properties (height, specific volume, and crust color) as the control bread. While the addition of hydrolysate decreased the hardness of the breads, it positively improved important starch fractions (increasing the amount of slowly digestible starch and decreasing the amount of rapidly digestible starch). It also increased antioxidant capacity (iron (III) reducing antioxidant power, ABTS, and DPPH (2,2‐diphenyl‐1‐picrylhydrazyl) and reduced the starch hydrolysis index of the bread. It was shown that the hydrolysate obtained after the hydrothermal treatment of bran could be used in bread making to satisfy the demand for products preferred by consumers from both health and sensory points of view.

## 1 INTRODUCTION

Bread is an important food item obtained by mixing and kneading wheat flour, yeast, and salt with certain proportions of water, and cooking it after fermentation for a certain period of time. It has a significant role in the human daily diet because it is cheap, satisfying, and easily available with a unique neutral aroma (Elgün & Ertugay, 1995). Today, developing and changing living conditions and increasing health problems have led people to consume healthy forms of bakery products (Foschia et al., 2013). So far, different ingredients such as wheat bran (Arte, 2019; Hemdane et al., 2016), organic acids (Su et al., 2019), phenolics (Pasrija et al., 2015), flour, or protein isolates of different legumes (chickpea, pea, pigeon pea, lentil, cowpea, etc.) (Hoque et al., 2022), barley, oat (Rieder et al., 2012), and sorghum flour (Dube et al., 2020), fruit extract (Ezhilarasi et al., 2013), pumpkin puree (Ebrahimi et al., 2022), marine green microalgae (*Tetraselmis chuii*) (Qazi et al., 2021), and grape seed byproducts (Elkatry et al., 2022) have been executed in flour to improve bread quality and increase its functionality.

Wheat bran (WB), rich in fiber and protein content, is stripped away from wheat grain during the rolling process and becomes a byproduct. Besides cellulose (30%), hemicellulose (25%), and lignin (8%) content (Urbaniec & Grabarczyk, 2009), it is rich in bioactive compounds and micronutrients (Katileviciute et al., 2019) such as phenolic acids (Yin et al., 2019), some vitamins and minerals (Ye et al., 2021). It is widely used in bread production, but it is known that the direct addition of wheat bran affects negatively dough rheology (Cingöz et al., 2023), bread properties (gluten dehydration, recrystallization of amorphous starch, or redistribution of water molecules among bread components), the production process, and thus bread quality (Fadda et al., 2014).

Consumers have a preference for bread with high volume, desired color and texture, and homogeneous crumb structure. The use of bran in bread making negatively affects the appearance and texture of bread. There have been different attempts to reduce or eliminate these negative effects including reducing the bran size (Cai et al., 2014; Cingöz et al., 2023; Kaprelyants et al., 2013; Noort et al., 2010), using enzymes (Yan et al., 2022), addition of vital gluten (Messia et al., 2016; Ortolan & Steel, 2017), fermentation (Hemdane et al., 2016), extrusion (Gomez et al., 2011), and hydrothermal treatment of wheat bran (Cingöz et al., 2023; Kabel et al., 2007). Some enzymes including α‐amylases (Liu et al., 2017), xylanase (Yan et al., 2022), cellulase (Park et al., 2018), lipase (Melis et al., 2019), dextransucrase (Kajala et al., 2015), protease (Fuentes et al., 2016), oxidative enzymes (Fuentes et al., 2016) affect dough stability and bread shelf life positively and reduce the rate of staling and thus prolong bread freshness. Fermentation of bran changes the cell wall structure by converting arabinoxylans into a soluble form and thus overcomes its negative effects on dough and bread. However, the removal of spent yeast from the bran could be a problem that causes a negative effect on the physical properties of the bread (Hemdane et al., 2016). Vital gluten addition could improve the rheological properties of flours with low protein content. However, it also has disadvantages such as higher production costs and an undesirable taste and odor in the products to which it is added (Ortolan & Steel, 2017). Wheat bran extrusion is a process in which chemical components such as dietary fiber contribute to the transition to a soluble form. However, besides changing the fiber structure the process also modifies starch, lipid, and protein components and reduces the nutritional and functional properties of the products (Gomez et al., 2011).

Alternatively, wheat bran can be treated by a hydrothermal process to overcome the negative effects of it on the dough and thus bread. The process is easy to apply and water is sufficient for hydrolysis of lignocellulosic materials (Garrote et al., 2002; Kabel et al., 2007). Processing at high temperatures in water causes autoionization of acids such as acetic and uronic acid in lignocellulosic material. This reaction increases the hydronium ion concentration (Vegas et al., 2006) and thus hemicellulose is hydrolyzed to soluble fractions such as oligosaccharides, monosaccharides, and sugar degradation products (furfural and hydroxymethylfurfural) while cellulose and lignin stay with the solid phases (Nagarani et al., 2014). The acidic environment also releases some phenolic compounds from hemicellulose and lignin (Conde et al., 2011) and increases the total phenolic matter content of the reaction medium (Cingöz et al., 2023).

In our literature search, we did not come across any study on the use of the wheat bran hydrolysate (WBH) in bread making. The aim of the present study was to investigate the effect of liquor obtained from the hydrothermal treatment of WB at 150°C on bread including processing, physicochemical (volume, weight, height, specific volume, color, dry matter, ash, protein and fat content) and nutritional properties (total phenolic and flavonoid substances, antioxidant activity and important starch fractions).

## 2 MATERIALS AND METHODS

### 2.1 Materials

Wheat flour (0.70 < ash % ≤ 0.80; wet gluten > 28%; gluten index > 60; protein > 10.5; water holding capacity >58%) and wheat bran (>850 μm) were provided by Birsan A.Ş. in Tokat, Türkiye, and stored at 4°C. Baker's yeast and hemicellulase (*Aspergillus oryzae*, Polenzyme × 1000 enzymes) were from Pak Maya Bakers' Yeast Co. (Izmir, Turkey) and Polen Gıda (Istanbul, Turkey), respectively. The glucose oxidase/peroxidase (GOPOD) assay kit and pancreatin (Porcine pancreas 4×USP) and invertase (3000 U/mL) were obtained from Biasis (Turkey) and Sigma‐Aldrich (St. Louis, MO, USA), respectively. Amyloglucosidase (3300 U/mL) was bought from Megazyme (Ireland).

### 2.2 Methods

#### 2.2.1 Hydrothermal treatment

Wheat bran (WB) was mixed with water at a ratio of 8:1 w/w (liquid/solid) and hydrolyzed at 150°C for 30 min in a 1 L stainless steel reactor (Parr Reactor, Co., IL, USA) (Cingöz et al., 2023). The spent solid of WBH was removed from the filtrate (Garrote et al., 1999) that was used for bread making.

#### 2.2.2 Bread making

Bread was made by the slightly modified method of The American Association of Cereal Chemists (AACC) Standard Method 10‐10.03 (AACC, 2004). Hydrolysates obtained from wheat bran were used in two forms with and without enzyme addition in the bread making. To make the bread without enzyme addition (WBH), hydrolysates were used directly in bread making while hemicellulase enzyme, 10 mg/100 mL, was added to the hydrolysates to make the bread with enzyme addition (WBHE). Both of them were added to the dough‐kneading water in five different ratios (10%, 20%, 30%, 50%, and 100%) based on water displacement. Water was added to the flour based on Mixolab water absorption value (58.9%). The dough was prepared and fermented for 60–20–60 min (first punching, second punching, and proofing) at 30 ± 1°C and 80 ± 5% relative humidity. The breads were baked in pre‐oiled special bread pans (10 × 13 × 5 cm) in a convection steam oven at 230 ± 5°C and 60% relative humidity (Kromlux KKF‐E/10, Turkey) for 20 min. They were cooled for 5 h after baking, packed in polyethylene bags, and used for the analysis.

#### 2.2.3 Physical analysis

The weights (g) and heights (cm) of all the bread were measured. AACC Standard Method No. 10‐05.01 (AACC, 2004) was used to determine their volumes. The specific volume of the bread was calculated by the following equation (Araki et al., 2009).

$$\mathrm{Specific}\;\mathrm{volume}=\mathrm{volume}\;\mathrm{of}\;\mathrm{bread}\;\left(\mathrm{mL}\right)/\mathrm{weig}\mathrm{ht}\;\mathrm{of}\;\mathrm{bread}\;\left(g\right)$$

#### 2.2.4 Chemical analysis

The moisture (AACC Standard Method No. 44‐01.01) and ash content (AACC Standard Method No. 08‐01.01) of the breads were determined (AACC, 2004). The micro‐Kjeldahl method was used to determine nitrogen content (AOAC, 2000). Crude fat was determined gravimetrically using Ankom XT10 extraction system (Ankom Technology Inc., Macedon) (AOCS, 2005).

#### 2.2.5 Color measurement

The color of the crumb and crust of the bread samples was measured with a Minolta CR300 (Minolta Inc., Tokyo, Japan) using the Hunter L*, a*, and b* color scales. The Δ*E* value was calculated according to Equation (1).

(1) $$\Delta E={\left[{\left(L-L_{\mathrm{ref}}\right)}^{2}+{\left(a-a_{\mathrm{ref}}\right)}^{2}+{\left(b-b_{\mathrm{ref}}\right)}^{2}\right]}^{1/2}$$

#### 2.2.6 Texture analysis

The texture measurements of the bread were carried out (V_test_: 35 mm/min, V_return_: 500 mm/min, V_pos1_: 500 mm/min, V_pos2_: 10 mm/min, L_max_: 10 mm, F_v_: 0.1 N) at 0, 24, 48, and 72 h after the production of bread. The hardness measurements of the bread were determined in N/cm^2^ according to Aydın and Öğüt (1991) using a texture analyzer (a round head of 2 cm diameter and at a pressure of 10 mm) (Zwick Z0.5, Germany) at different time intervals.

#### 2.2.7 Phenolic and flavonoid content and antioxidant capacity analysis

Produced breads with WBH and WBHE were subjected to an extraction procedure to determine total phenolic content and total flavonoid content. The extraction was carried by mixing (200 rpm, 1 h, room temperature) 25 g of sample with 50 mL of 50% acetone. The extract was separated by filtration and this procedure was repeated three times. All the extracts were combined, centrifuged at 2500 × *g* (4500 rpm in M2 rotor, Boeco U‐32R, Germany) for 10 min and evaporated at 45°C until around 10 mL supernatant was obtained. The volume of final solutions was made up to 25 mL with distilled water and stored at −18°C until use (Eberhardt et al., 2000).

The phenolic and flavonoid content of the extracts was measured by the Folin–Ciocalteu method using gallic acid as a standard (Singleton & Rossi, 1965) and by the method provided by Li et al. (2015) using quercetin standard, respectively. Trolox equivalent (TE) antioxidant capacity 2,2'‐azino‐bis‐3‐ethylbenzthiazoline‐6‐sulphonic acid (ABTS) (Re et al., 1999), iron (III) reducing antioxidant power (FRAP) (Benzie & Strain, 1996), and 2,2‐diphenyl‐1‐picrylhydrazyl (DPPH) radical scavenging activity (Brand‐Williams et al., 1995) methods were used to determine the antioxidant capacities of the extracts and the results were expressed as TE.

#### 2.2.8 Determination of important starch fractions

Total glucose (TG), rapidly available glucose (RAG), total starch (TS), rapidly digestible starch (RDS), slowly digestible starch (SDS), free glucose (FG), and starch hydrolysis index (SHI) of bread were determined by in vitro digestion method (Englyst et al., 1992). The ground samples, 1.00 ± 0.01 g, were placed in 15 mL glass tubes, and 50 mg of guar gum, 15 pieces of 4 mm glass beads, and 4 mL of acetate buffer (0.5 M, pH 5.2, and 5 mM CaCl_2_) were added to the tubes and vortexed for 30 s. To the mixture, 1 mL of the hydrolytic enzyme mixture (0.90 g of pancreatin‐Sigma P7545 dissolved in 4 mL of water) was added and mixed in a magnetic stirrer for 10 min. Centrifugation was then performed at 1500 × *g* (3500 rpm in M2 rotor, Boeco U‐32R, Germany) for 10 min and the supernatant was separated. Then 2.7 mL of the pancreatin supernatant was taken, mixed with 0.3 mL of amyloglucosidase (0.32 mL of amyloglucosidase was diluted with 0.4 mL of distilled water), and 0.2 mL of invertase (10 mg/mL) and shaken in a water bath at 37°C in a horizontal position at 160 rpm. After 20 (G20) and 120 (G120) min, 0.2 mL of the sample was removed from the tubes and transferred to tubes containing 4 mL of 95% ethanol to stop the reaction (G20, G120). G20 and G120 fractions were centrifuged at 4000 × *g* (5700 rpm in M2 rotor, Boeco U‐32R, Germany) for 10 min and glucose amounts in their supernatants were determined by GOPOD method.

For the determination of FG, the steps, described above, were followed but only invertase enzyme was used. Invertase, 0.2 mL (50 mg of 300 U/mg invertase was weighed and dissolved in 5 mL distilled water) was added to the tubes and shaken in a shaking water bath at 37°C at 160 rpm horizontal position for 120 min. At 120 min, 0.2 mL of the sample was taken and transferred to tubes containing 4 mL of 95% ethanol, vortexed, and the reaction was stopped. The tubes were then centrifuged at 4000 × *g* (5700 rpm in M2 rotor, Boeco U‐32R, Germany) for 10 min, and the amount of glucose in the supernatants was determined by the GOPOD method to find FG.

$$\mathrm{TS}=\left(\mathrm{TG}-\mathrm{FG}\right)\times 0.9$$

RAG=G20

$$\mathrm{RDS}=\left(G20-\mathrm{FG}\right)\times 0.9$$

$$\mathrm{SDS}=\left(G120-G20\right)\times 0.9$$

$$\mathrm{SHI}=\left(\mathrm{RDS}/\mathrm{TS}\right)\times 100$$

#### 2.2.9 Statistical analysis

The analysis of variance (ANOVA) of the results was performed by using SPSS statistical program (SPSS, Inc., Chicago, IL, USA), and Duncan multiple comparison test was employed to evaluate the differences between groups with a 95% confidence interval.

## 3 RESULTS AND DISCUSSION

In this study, an alternative method was employed to eliminate the negative effects of wheat bran addition in bread making. WB was hydrolyzed at 150°C for 30 min, and the hydrolysates obtained were used in bread making according to the method provided by Cingöz et al. (2023). The previous study reported that the hydrolysate obtained at 150°C could be used in bread making without adversely affecting the rheological properties of the dough (Cingöz et al., 2023). The WBH obtained had 5.18% dry matter, 0.63 g/L glucose, 0.98 g/L xylose, 2.40 g/L xylooligosaccharide, and 1156.84 µg GA/mL total phenolic content. It was added to the dough‐kneading water in various proportions and bread making was carried out.

The results of the chemical analysis of the bread samples are shown in Table 1. The addition of WBH resulted in bread with high water absorption capacity. The dry matter content of bread varied from 32.36% to 34.22% with the addition of WBH and 32.32% to 33.89% with the addition of WBHE. The high water absorption capacity of bread with WBH and WBHE addition decreased their dry matter content them. It was found that the addition of WBH increased the ash content by 2.22% to 3.06%, while the ash content of the WBHE ranged from 0.91% to 1.56%. No change was observed in the total fat and total protein content of the bread made with the addition of WBH and WBHE.

**TABLE 1:** Physical and chemical analysis results of bread

Samples	Volume (cm^3^)	Weight (g)	Height (cm)	Specific Volume (cm^3^/g)	Dry matter (%)	Ash (%)	Protein (%)	Fat (%)
**CONTROL**	455.0±12.12^d^	98.3±0.57^e^	7.74±0.08^bc^	4.61	34.57±0.53^ab^	1.58±0.00^fg^	11.36±0.15^d^	0.63±0.03^a^
**10% WBH**	455.0±12.12^d^	100.3±0.57^cd^	7.72±0.03^bc^	4.53	32.85±0.41^fg^	2.22±0.01^e^	11.52±0.12^cd^	0.63±0.05^a^
**20% WBH**	479.7±5.77^c^	100.0±0.00^d^	7.64±0.05^c^	4.79	32.36±0.18^f^	2.64±0.11^d^	11.50±0.13^cd^	0.64±0.07^a^
**30% WBH**	502.7±6.11^ab^	100.3±0.11^cd^	7.87±0.06^a^	5.01	32.97±0.23^ef^	2.75±0.04^c^	11.53±0.23^cd^	0.67±0.02^a^
**50% WBH**	486.3±5.77^c^	100.0±1.00^d^	7.83±0.08^ab^	4.86	32.47±0.38^f^	2.93±0.03^b^	11.56±0.43^bcd^	0.68±0.03^a^
**100% WBH**	430.3±10.50^e^	102.0±0.00^ab^	7.01±0.03^fg^	4.21	34.22±0.14^bc^	3.06±0.02^a^	11.49±0.19^cd^	0.70±0.02^a^
**10% WBHE**	431.0±10.53^e^	100.0±1.00^d^	7.10±0.05^f^	4.51	32.32±0.39^f^	0.91±0.01^k^	11.52±0.04^cd^	0.68±0.05^a^
**20% WBHE**	493.3±10.50^bc^	100.3±0.57^cde^	7.23±0.07^e^	4.91	33.89±0.65^de^	1.16±0.02^j^	11.45±0.23^cd^	0.63±0.05^a^
**30% WBHE**	498.7±5.77^c^	100.6±1.15^bcd^	7.30±0.05^e^	4.95	33.52±1.00^cde^	1.28±0.00^ı^	11.59±0.07^abcd^	0.66±0.07^a^
**50% WBHE**	511.0±6.08^a^	100.3±1.52^cd^	7.53±0.06^d^	5.09	33.26±0.51^de^	1.43±0.02^h^	11.63±0.10^abcd^	0.63±0.04^a^
**100% WBHE**	430.7±10.50^e^	100.3±0.57^cd^	6.97±0.11^g^	4.29	32.64±0.38^ef^	1.56±0.00^f^	11.51±0.11^cd^	0.64±0.04^a^

The letters *a, b, c, … indicate statistical differences at the p*<*0.05 level of the samples in the same column.

The highest volume and height were found in the control bread and the breads made with 10%–50% hydrolysate addition (Table 1). The addition of 100% WBH causes a partial decrease in the bread volume. Similar results were found in breads made from WBHE. During the fermentation process of the dough, baker's yeast consumes the glucose in the environment and creates CO_2_. The resulting CO_2_ causes the bread to rise. The more reducing sugar are produced that yeast could use, the more CO_2_ are produced (Zhang et al., 2005). Therefore, the reducing sugar of WBH (0.63 g/L glucose) supported yeast action during the fermentation phase and positively affected the volume of bread. Despite more reducing sugar content of 100% WBH and WBHE, both had more soluble hemicellulose which could enter into the gluten network structure and disrupt the structure (Sui et al., 2018) resulting in a decrease in the bread volume.

The value of specific volume of the breads varies between 4.21 and 5.09 cm^3^/g (Table 1). These values increased up to 50% WBH addition and decreased at 100% WBH addition. It can be seen that a similar situation occurs in breads with WBHE. It has been reported in the literature that the direct addition of WB to the flour reduced the bread volume (Cingöz et al., 2022; Gomez et al., 2011). In addition, bread made with high soluble fiber such as inulin, carob fiber, pea fiber, and jackfruit were reported to have lower volume than bread without added fiber (Feili et al., 2013) and millet flour (Patil et al., 2016). Studies found that the coarse or medium size of bran addition to bread had an effect on bread volume negatively (Alzuwaid et al., 2021; Cingöz et al., 2022; Curti et al., 2013, Kim et al., 2013; Rezaei et al., 2019). When compared with the literature, it is clear that the addition of hydrolysate improves the physical properties of the bread by increasing the specific volume value, and this feature causes an advantage in bread production compared to the studies in the literature.

**FIGURE 1:**
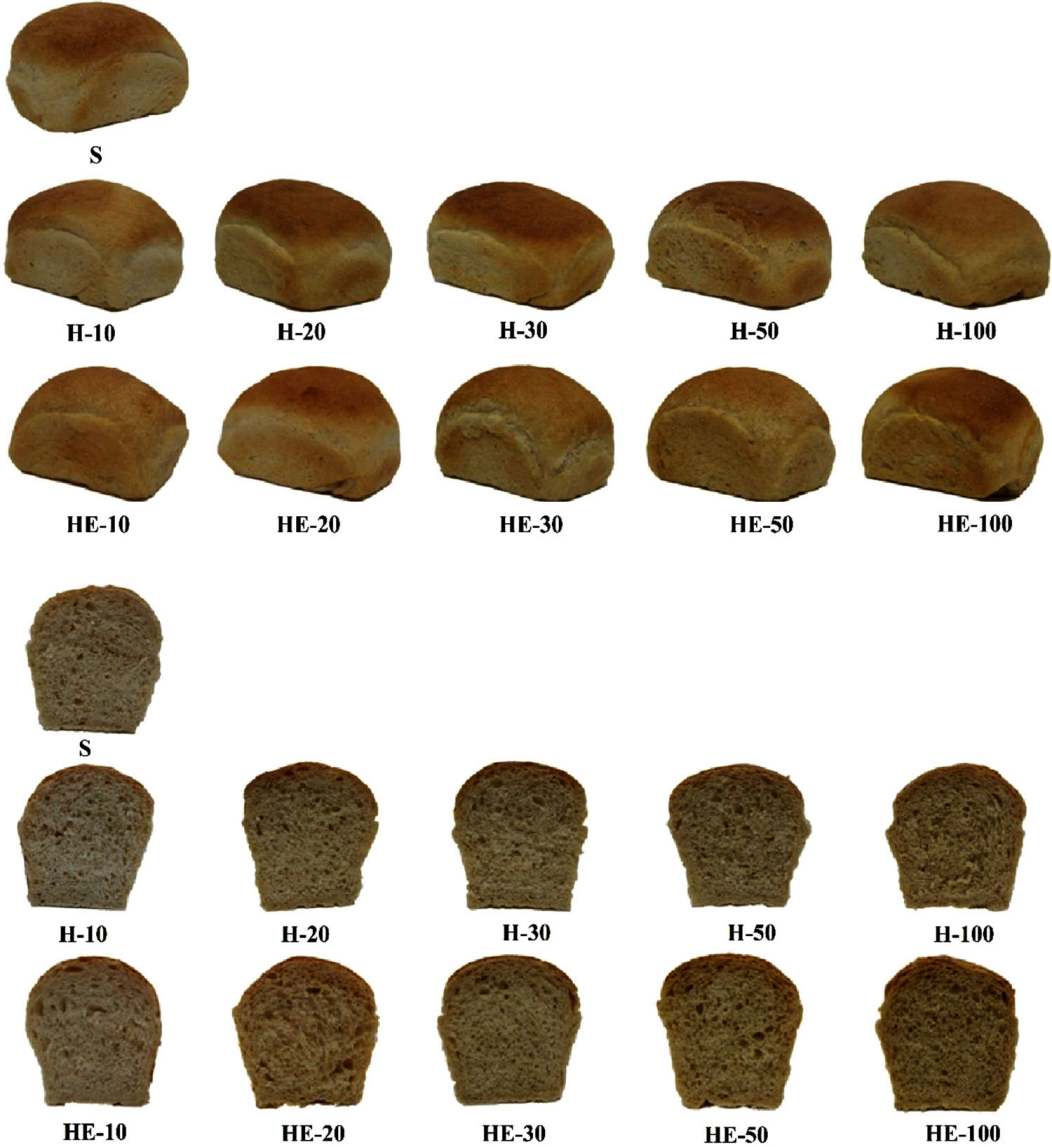
The crust and crumb pore structures of breads. (S: Control, H‐10: 10% wheat bran hydrolysate [WBH] was added, H‐20: 20% WBH was added, H‐30: 30% WBH was added, H‐50: 50% WBH was added, H‐100: 100% WBH was added, HE‐10: 10% wheat bran hydrolysate with added enzyme [WBHE] was added, HE‐20: 20% WBHE was added, HE‐30: 30% WBHE was added, HE‐50: 50% WBHE was added, HE‐100: 100% WBHE was added).

Figure 1 presents the crust of the breads and the pore structures of the crumb. Bread with a yellow crust color, homogeneous pore structure, and a light yellow are desirable properties for consumers (Castro et al., 2017). The use of bran in bread making darkens the crust and crumb color and negatively affects consumer preferences (Rezaei et al., 2019). It was found that the addition of WBH had no negative effect on the crust appearance, the color of the crumb went toward yellow and the pore structure was not adversely affected thus the darkening effects caused by the use of bran on the color could be eliminated. The addition of WBHE was found to have a similar effect. The color values of crust and crumb of all bread samples are shown in Table 2. The *L*, a*, and b* values of crusts varied between 61.84 and 66.51, 7.96 and 12.23, and 30.81 and 36.55, respectively, while Δ*E* values that donated the total color difference were between 3.22 and 5.62. The low Δ*E* indicates that the bread samples are close to the control in terms of total color values. As the amount of hydrolysate addition increased, the Δ*E* values of the breads also increased. Δ*E* increase was also observed in the enzyme added samples. It was found that the addition of hydrolysate caused a decrease in L* values compared to the control bread. The bread crust a* values increased significantly with the addition of hydrolysate (*p* < 0.05). The highest a* value of the crust (12.23) was observed in breads with 30% WBHE added. The addition of hydrolysate also increased the b* crust values of the breads, and the results ranged from 31.86 to 34.17. Similar results were observed when the enzyme together with the hydrolysate was added.

**TABLE 2:** Color properties of bread

Samples	CRUST				CRUMB			
	L	a	b	ΔE	L	a	b	ΔE
**CONTROL**	65.30±2.00^ab^	7.96±0.19^fg^	30.81±0.71^d^	‐	75.45±0.78^a^	‐1.01±0.01^ef^	14.41±0.15^ef^	‐
**10% WBH**	63.45±0.72^cde^	8.45±0.89^ef^	33.92±1.25^b^	3.22	67.48±0.50^cde^	‐1.30±0.10^h^	14.41±0.15^ef^	7.90
**20% WBH**	64.04±0.12^bc^	9.16±0.22^d^	34.17±0.87^b^	3.65	67.44±0.35^de^	‐1.18±0.05^gh^	13.97±0.30^g^	7.55
**30% WBH**	63.83±1.15^cd^	8.86±0.34^de^	34.12±0.50^b^	3.73	68.05±0.21^c^	‐0.90±0.08^de^	14.12±0.09^fg^	7.06
**50% WBH**	61.84±0.54^fg^	9.12±0.14^d^	33.26±0.33^b^	4.40	67.84±0.20^cd^	‐0.88±0.07^de^	14.36±0.23^ef^	7.34
**100% WBH**	61.90±0.09^fg^	9.79±1.00^c^	31.86±0.50^c^	4.62	66.83±0.40^fg^	0.23±0.06^a^	16.63±0.20^b^	8.75
**10% WBHE**	62.14±0.33^efg^	8.87±0.12^de^	33.87±0.41^b^	5.36	61.69±0.40^h^	‐1.10±0.02^fg^	13.33±0.33^h^	7.38
**20% WBHE**	66.51±0.41^a^	10.15±0.28^c^	36.55±0.07^a^	5.62	68.86±0.32^b^	‐0.66±0.02^b^	15.44±0.35^d^	7.58
**30% WBHE**	64.16±0.17^bc^	12.23±0.03^a^	33.46±0.47^b^	5.15	66.38±0.50^g^	‐0.82±0.01^cd^	13.48±0.34^h^	8.63
**50% WBHE**	63.46±0.12^cde^	11.84±0.21^ab^	34.24±0.22^b^	5.50	67.19±0.36^ef^	‐0.73±0.01^bc^	16.04±0.03^c^	8.15
**100% WBHE**	62.38±0.60^ef^	11.56±0.42^b^	31.96±0.03^c^	5.36	67.55±0.17^cde^	0.29±0.03^a^	17.93±0.01^a^	8.58

The letters *a, b, c, … indicate statistical differences at the p*<*0.05 level of the samples in the same column.

The color characteristics of the bread crumb structures are shown in Table 2. The L* value, which was 75.45 for the control bread, was determined to be 66.83 for the bread produced with 100% WBH. Similar results were observed for the enzyme added bread. The a* value of the crumb, which was −1.01 in the control bread, increased with the addition of WBH and WBHE. The a* values of the breads with 30% WBH and 10% WBHE addition were statistically similar to the control bread. The b* color values of the crumb of breads with WBH addition were statistically similar to those of the control breads. The highest b* values of the crumbs were observed in bread produced with 100% WBH and WBHE. Up to 50% WBH addition, the color differences in bread crumbs decreased, while they increased when 100% WBH was added. A previous study reported that the addition of 10%, 20%, and 30% WB to wheat flour caused undesirable color in the crust and crumb color of bread, the addition of more than 10% of WB increased the hardness of bread (Cingöz et al., 2022). In this study, it was found that the color differences of the breads produced with 30% WBH and 20% WBHE were closest to that of control bread. The addition of WBH changed the crumb color of the bread to a darker yellow.

**FIGURE 2:**
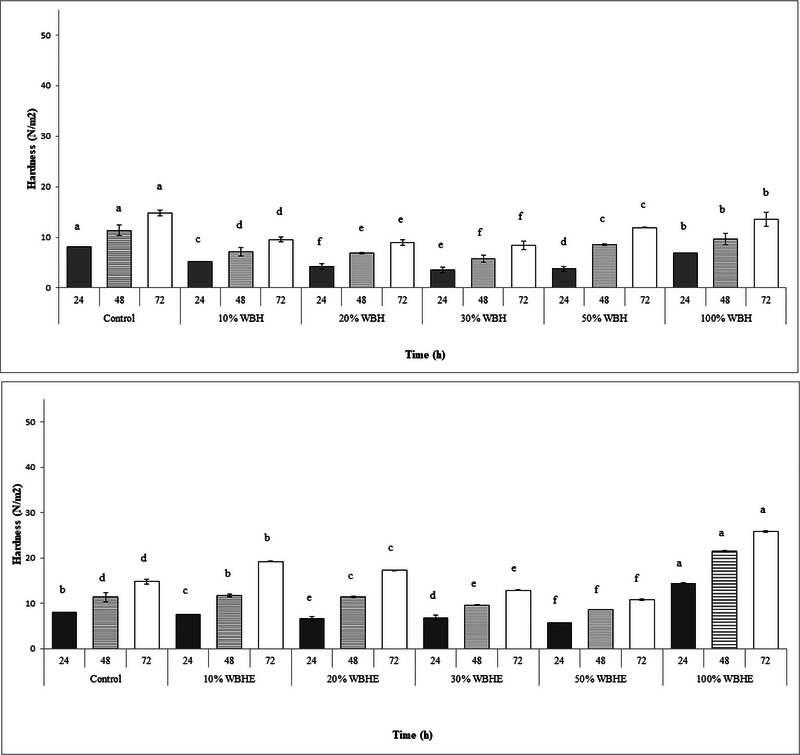
Hardness (N) values (%) of hydrolyzate added bread and enzyme‐treated hydrolyzate added bread. The letters a, b,c.... indicate the statistical differences of the samples in the columns at the same time period at p<0.05 level. (WBH: bread with added wheat bran hydrolysate; WBHE: bread with enzyme added wheat bran hydrolysate)

It is believed that the main reason for bread staling is the retrogradation of starch. However, studies have shown that starch retrogradation is not the only factor for staling. Surface drying and binding between starch and gluten are also effective in the staling phenomena (Martin,, 1991). Texture measurements during bread production and storage are an important parameter for measuring not only bread quality but also to get an idea of the staling of bread (Lassoued et al., 2008). The increases in the hardness of bread were determined with the texture analyzer for 24, 48, and 72 h after baking and the results are shown in Figure 2. The addition of WBH up to 50% decreased the hardness of the crumb. The hardness value of the control bread increased from 3.58 to 14.81 Nm after 72 h. Hardness values decreased in WBH added breads compared to control bread. A similar situation is also observed for breads with WBHE addition. The addition of hydrolysate to the dough‐kneading water caused the bread to remain softer due to the increase in the water holding capacity of the hydrolysates. It was reported that the addition of dietary fiber (Jensen et al., 2015) and coarse and fine bran (Curti et al., 2013; Le Bleis et al., 2015) to flour progressively increased the initial firmness value of breads. On the other hand, the addition of potato flour to bread was reported to reduce the hardness of it (Meng et al., 2022), which was similar to the results of this study.

### 3.1 Functional properties

The health benefits of phenolics are well known including anti‐inflammatory, antioxidant, antimicrobial, and protein/enzyme neutralization/modulation mechanisms (Ozcan et al., 2014). The total phenolic substances, total flavonoid content, and the antioxidant capacity of the bread samples were analyzed and the results are presented in Table 3. The total phenolic content was 21.14 mg GA/100 g for the control bread, increased to 39.64 mg GA/100 g for the bread produced with 100% WBH addition and to 57.98 mg GA/100 g for the bread produced with 100% WBHE addition. The total flavonoid content of bread increased from 10.78 to 22.88 QE/100 g with 100% WBH addition and 30.49 QE/100 g with 100% WBHE addition.

**TABLE 3:** Total antioxidant capacity, total flavonoid and total phenolic content results of bread

Samples	Total Flavonoid Content (μg QE/100 g)	Total Phenolic Content (mg GA/100 g)	Antioxidant Activity		
			DPPH•	ABTS•+	FRAP
			(μM TE/100 g)		
**CONTROL**	10.78±0.52^h^	21.14±1.70^g^	1.12±0.09^fh^	4.22±0.11^f^	2.25±0.02^ı^
**10% WBH**	9.92±0.35^m^	25.89±0.29^f^	1.10±0.07^fh^	4.48±0.27^f^	2.73±0.16^h^
**20% WBH**	11.07±0.06^h^	24.81±0.46^f^	1.28±0.06^gh^	5.54±0.12^e^	3.33±0.02^g^
**30% WBH**	12.82±0.26^g^	33.10±3.42^e^	1.43±0.03^efg^	6.48±0.08^e^	3.68±0.05^f^
**50% WBH**	17.25±0.32^e^	33.43±0.17^e^	1.75±0.06^d^	7.66±0.02^c^	4.24±0.02^de^
**100% WBH**	22.88±0.26^b^	39.64±0.13^bc^	2.07±0.07^b^	8.91±0.05^b^	5.26±0.12^b^
**10% WBHE**	14.14±0.15^f^	38.48±1.13^c^	1.59±0.14^de^	7.13±0.56^d^	4.04±0.20^e^
**20% WBHE**	14.98±0.69^j^	36.64±0.04^cd^	1.61±0.06^cde^	7.29±0.02^d^	4.28±0.00^de^
**30% WBHE**	18.17±0.49^d^	35.31±0.54^de^	1.62±0.02^cde^	7.17±0.12^d^	4.23±0.04^de^
**50% WBHE**	20.98±0.61^c^	36.89±0.88^cd^	1.56±0.24^ef^	7.91±0.10^c^	4.63±0.02^c^
**100% WBHE**	30.49±0.06^a^	57.98±3.21^a^	2.13±0.02^ab^	9.75±0.07^a^	5.61±0.63^a^

The letters *a, b, c, … indicate statistical differences at the p*<*0.05 level of the samples in the same column.

The antioxidant activities of the breads were determined by three different methods and their antioxidant capacities were determined in the range of 1.10–2.13 µM TE/100 g by the DPPH method, 4.22–9.75 µM TE/100 g by the ABTS method, and 2.25–5.61 µM TE/100 g by the FRAP method. It can be seen that depending on the amount of hydrolysate addition the antioxidant capacities of the bread increase. The addition of the WBHE increased the total flavonoid, total phenolic, and total antioxidant capacities of the breads.

The previous study reported that the total phenolic content of bread made with 100 g of wheat flour was found as 20.83 mg GA/100 g and that of breads made with millet in three different ratios (10%, 20%, and 30%) ranged from 29.59 to 80.74 mg GA/100 g (Patil et al., 2016). Yu et al. (2013) found that the total phenolic content of white bread ranged from 0.79 to 1.03 mg/g and that of whole wheat bread ranged from 1.50 to 1.65 mg/g for bread. Chlopicka et al. (2012) showed the total flavonoid of bread from wheat flour as 20.30 µg catechin per gram. It was reported that whole grain flour had higher flavonoid content than refined flour (Li et al., 2015) and fine bran contains more flavonoid substances than coarse bran (Brewer et al., 2014). Lavelli et al. (2009) measured the antioxidant activity of bread made with wheat flour using the DPPH method and determined it to be 0.58–1.46 µmol TE/g. Liu et al. (2010) determined the antioxidant activity of DPPH in six different wheat breads ranging from 6.48 to 8.57 µmol TE/g. In this study, the total phenolic content of the bread (21.14–57.98 mg GA/100 g) coincided with the literature while their antioxidant capacities of them were found to be higher than the reported values in this study.

### 3.2 Important starch fractions

Although starch occupies an important place in the human diet, excessive bread consumption due to its high starch content is associated with several health problems (such as obesity, diabetes, and cardiovascular disease). In vitro starch digestion rate groups starch into three namely RDS, SDS, and resistant starch (RS) (Aarathi et al., 2003; Dona et al., 2010; Englyst et al., 1992; Venn & Mann, 2004). Consumption of food with a high content of RAG causes a rapid increase in blood glucose levels, disrupting the sugar regulation of metabolism (Ergun, 2014) and foods with low glycemic index have lower RAG values (Thorsdottir & Birgisdottir, 2005). In the context of prevention and control of metabolic diseases, it is important to choose foods with a high content of SDS and a low content of RDS (Venn & Mann, 2004). The nutritionally important starch fractions of the bread samples produced in this study were determined and the results are shown in Table 4.

**TABLE 4:** The content of important starch fractions and predicted glycaemic index of bread

Samples	RAG (%)	RDS(%)	SDS(%)	SHI
**CONTROL**	41.54±0.39^a^	37.39±0.35^a^	3.86±0.12^ı^	82.17±1.37^a^
**10% WBH**	40.22±0.05^b^	36.19±0.05^b^	5.30±0.38^gh^	79.78±0.49^b^
**20% WBH**	37.08±0.13^c^	33.37±0.12^d^	6.82±0.46^f^	73.78±0.28^c^
**30% WBH**	34.99±0.13^e^	31.49±0.12^e^	9.73±0.43^d^	69.84±0.77^d^
**50% WBH**	33.01±0.04^f^	29.71±0.04^f^	10.80±0.19^bc^	66.29±0.56^e^
**100% WBH**	30.37±0.09^l^	27.33±0.08^g^	13.67±0.12^a^	61.91±0.63^f^
**10% WBHE**	39.99±0.13^b^	35.99±0.12^b^	4.85±0.04^h^	79.33±0.83^b^
**20% WBHE**	37.47±0.09^d^	33.73±0.08^c^	7.02±0.43^ef^	74.56±0.72^c^
**30% WBHE**	34.60±0.09^e^	31.14±0.08^e^	10.39±0.16^c^	69.06±0.68^d^
**50% WBHE**	32.71±0.22^g^	29.44±0.20^f^	11.34±0.04^b^	65.68±0.91^e^
**100% WBHE**	29.67±0.22^h^	26.71±0.20^h^	13.46±0.43^a^	60.51±0.88^f^

*Note*: The letters a, b, c, … indicate statistical differences at the *p* *<* 0.05 level of the samples in the same column.

Abbreviations: RAG, rapidly available glucose (RAG); RDS, rapidly digestible starch; SDS, slowly digestible starch; SHI, starch hydrolysis index

The RAG content was 41.54% in the control bread, decreased with the addition of hydrolysate and it was 30.37% for the bread produced with 100% hydrolysate. A similar situation was observed in breads with WBHE addition, and 29.67% RAG was determined for the bread produced with 100% WBHE addition. It is clearly seen that WBH addition decreases RAG that is a desirable property in terms of health. Compared to the control bread, SDS increased 3.54‐fold and 3.49‐fold for the bread produced with 100% WBH and 100% WBHE, respectively. On the other hand, RDS decreased to 27.33% and 26.71% for the bread with 100% WBH and 100% WBHE, respectively.

The ratio of RDS to total starch in foods is defined as the SHI. It relatively reflects the in vitro digestion rate of starch in foods, as well as the GI value (Englyst et al., 2003). It was determined that the addition of hydrolysate decreased the SHI value, and this decrease was parallel to the increase in the amount of added hydrolysate. The SHI value, which was 82.17 for the control bread, decreased to 61.91 for the bread produced with 100% WBH addition and to 60.51 for the bread produced with 100% WBHE addition. Since the hydrolysis of starch is slowed down by the gluten matrix surrounding the starch granules and the arabinoxylan component, studies have shown that starch hydrolysis of wheat products with high arabinoxylan is slower (Bravo et al., 1998). The hydrothermal processes released the soluble arabinoxylans from the structure of bran and slowed the hydrolysis of starch which could have the potential to lower the glycemic index (GI) of foods. Glycemic index is a concept that expresses the effect of carbohydrates on blood sugar levels and a high glycemic index (GI) diet increases the risk of many health problems including diabetes, cardiovascular disease, and mortality (Jenkins et al., 2021). It was reported that the increased soluble fiber content in a food lowered the glycemic index value of value (Demirkesen‐Bicak et al., 2021). Since the addition of hydrothermally processed WB to bread slowed down the hydrolysis of starch, it is predicted that the decrease in SHI values will also cause a decrease in GI values of breads.

The study about different bread types in South and Central Asia reported that the RDS ratio decreased and the SDS ratio increased with the introduction of components such as bran and dietary fiber into breads (Ranawana & Henry, 2013). In the study on starch fractions of breads in Turkey, 68.0% fast digestible starch and 2.0% slow digestible starch were determined in wheat bran breads (Taş & El, 2000). A previous study reported that the use of wheat bran in bread production caused a decrease in the amount of RDS and an increase in the amount of SDS as well as a reduction in SHI values (Cingöz et al., 2022). Studies reported that the addition of oat bran and oat beta‐glucan decreased the in vitro GI (Hu et al., 2022) and the addition of small millet flour (Sharma & Gujral, 2019) and plant sterols (Makran et al., 2022) increased the slow digestible starch content of breads. The study about the effects of soluble and insoluble dietary fiber additions on the digestion rate of starch reported that as the soluble dietary fiber content in the samples increased, the amount of RDS decreased and the amount of SDS increased (Oh et al., 2014). As a result, the soluble dietary fiber content of the hydrolysates used in bread production resulted in high SDS and low RDS results in breads.

## 4 CONCLUSION

In this study, an alternative method was proposed instead of the direct addition of WB in bread making to eliminate the technological problems caused by the direct WB addition. Hydrothermally treated WB was used in the bread production and the addition of up to 50% WBH to the dough‐kneading water improved the physical properties of the breads. The dark color of the bread after the addition of wheat bran was eliminated. Also it reduced the hardness and improved important starch fractions positively (an increase in the amount of SDS and a decrease in the amount of RDS and SHI values), and antioxidant capacity (FRAP, ABTS, and DPPH) of the breads. The decrease in RDS and SHI values decreased the glycemic index values of the breads. With the use of WBH in bread making, it seems possible to reduce technological problems such as low bread volume, undesirable crust and crumb color, unusual taste and odor, cracking, and crust detachment in the crust. The results of this study showed that the hydrolysate obtained from the hydrothermally treated bran can be used in bread making to satisfy the demand for products that consumers prefer both in terms of health and senses.

## AUTHOR CONTRIBUTIONS

**Ali Cingöz**: Writing—original draft; methodology; writing—review and editing; visualization; project administration. **Özlem Akpinar**: Writing—review and editing; formal analysis. **Abdulvahit Sayaslan**: Formal analysis; writing—review and editing.

## CONFLICT OF INTEREST STATEMENT

The authors confirm that they have no conflicts of interest with respect to the work described in this manuscript.
